# A database for evaluating the InMAP, APEEP, and EASIUR reduced complexity air-quality modeling tools

**DOI:** 10.1016/j.dib.2019.104886

**Published:** 2019-11-28

**Authors:** Kirk R. Baker, Meredith Amend, Stefani Penn, Joshua Bankert, Heather Simon, Elizabeth Chan, Neal Fann, Margaret Zawacki, Ken Davidson, Henry Roman

**Affiliations:** a U.S. Environmental Protection Agency, Research Triangle Park, NC, USA; b Industrial Economics, Incorporated, Cambridge, MA, USA; c U.S. Environmental Protection Agency, Ann Arbor, MI, USA

**Keywords:** InMAP, APEEP, EASIUR, CAMx, CMAQ, BenMAP, PM2.5

## Abstract

Policy analysts and researchers often use models to translate expected emissions changes from pollution control policies to estimates of air pollution changes and resulting changes in health impacts. These models can include both photochemical Eulerian grid models or reduced complexity models; these latter models make simplifying assumptions about the emissions-to-air quality relationship as a means of reducing the computational time needed to simulate air quality. This manuscript presents a new database of photochemical- and reduced complexity-modelled changes in annual average particulate matter with aerodynamic diameter less than 2.5 μm and associated health effects and economic values for five case studies representing different emissions control scenarios. The research community is developing an increasing number of reduced complexity models as lower-cost and more expeditious alternatives to full form Eulerian photochemical grid models such as the Comprehensive Air-Quality Model with eXtensions (CAMx) and the Community Multiscale Air Quality (CMAQ) model. A comprehensive evaluation of reduced complexity models can demonstrate the extent to which these tools capture complex chemical and physical processes when representing emission control options. Systematically comparing reduced complexity model predictions to benchmarks from photochemical grid models requires a consistent set of input parameters across all systems. Developing such inputs is resource intensive and consequently the data that we have developed and shared (https://github.com/epa-kpc/RFMEVAL) provide a valuable resource for others to evaluate reduced complexity models. The dataset includes inputs and outputs representing 5 emission control scenarios, including sector-based regulatory policy scenarios focused on on-road mobile sources and electrical generating units (EGUs) as well as hypothetical across-the-board reductions to emissions from cement kilns, refineries, and pulp and paper facilities. Model inputs, outputs, and run control files are provided for the Air Pollution Emission Experiments and Policy Analysis (APEEP) version 2 and 3, Intervention Model for Air Pollution (InMAP), Estimating Air pollution Social Impact Using Regression (EASIUR), and EPA's source apportionment benefit-per-ton reduced complexity models. For comparison, photochemical grid model annual average PM_2.5_ output is provided for each emission scenario. Further, inputs are also provided for the Environmental Benefits and Mapping Community Edition (BenMAP-CE) tool to generate county level health benefits and monetized health damages along with output files for benchmarking and intercomparison. Monetized health impacts are also provided from EASIUR and APEEP which can provide these outside the BenMAP-CE framework. The database will allow researchers to more easily compare reduced complexity model predictions against photochemical grid model predictions.

**Table:** Specifications Table

Subject	Atmospheric Science
Specific subject area	Regional scale air quality modeling of chemically speciated particulate matter
Type of data	Table Figure
How data were acquired	The data was generating using software tools.
Data format	Raw Analysed
Parameters for data collection	Model inputs were developed for reduced form models recently published in peer reviewed literature
Description of data collection	The data includes model inputs and simulation configuration information for multiple reduced form models
Data source location	Institution: U.S. Environmental Protection Agency City/Town/Region: Research Triangle Park Country: USA
Data accessibility	Repository name: github Data identification number: Direct URL to data: https://github.com/epa-kpc/RFMEVAL

**Table:** 

**Value of the Data** • The dataset provided in this article will make it easier for researchers to compare multiple reduced complexity models against full-scale photochemical models using consistent inputs. • The dataset includes all necessary inputs (i.e., emission changes, meteorological data, and atmospheric chemistry) needed to run each reduced complexity tool. • This information can be used to replicate an existing evaluation and evaluate newer versions of these tools.

## 1 Data description

Regulatory assessments and research applications often use models to translate expected emissions changes from pollution control policies to estimates of air pollution changes and resulting changes in health impacts. Two approaches are typically used to simulate primary emitted and secondarily formed PM_2.5_ in the atmosphere: “full form” photochemical modeling and reduced complexity modeling. Full form photochemical modeling captures the complexities of environmental processes (e.g., atmospheric chemical reactions, gas-particle partitioning, dispersion of pollutants, and deposition to surfaces) by including detailed representations of each mechanism in the atmosphere to quantify the relationship between emissions and ambient concentrations. Local to regional scale dispersion of emissions is affected by many factors including emissions release characteristics (e.g., height above ground) as well as local topography and meteorological variables such as temperature and wind speed. In contrast, reduced complexity models use various methods to approximate estimates from full-scale photochemical models without explicitly representing the atmospheric chemical and physical processes that impact pollutant fate and transport.

This dataset includes inputs and outputs representing 5 emission control scenarios for multiple reduced complexity and full form models. Each emissions control scenario includes a projected future year reference and control scenario set of emissions. Inputs for each of the modeling systems were developed where possible with the same domain structure (e.g., grid cell size, domain extent, and vertical structure), reference emissions, emissions changes, and meteorology to facilitate comparison. In some situations, certain model formulations precluded implementation of consistency for certain inputs and those are noted in the following sections.

Here, a dataset (https://github.com/epa-kpc/RFMEVAL) is provided to help researchers perform systematic comparison of multiple reduced complexity models. The dataset includes consistent emissions for 5 different emissions control scenarios. Inputs were developed for 4 reduced complexity tools including the Intervention Model for Air Pollution (InMAP) [1], Air Pollution Emission Experiments and Policy Analysis (APEEP) versions 2 and 3 [2], Estimating Air pollution Social Impact Using Regression (EASIUR) [3], and EPA's source apportionment benefit-per-ton tool (SA-BPT) [4] (Table 1). Input files (emissions and where possible meteorology), output files (estimated changes in PM_2.5_ concentrations and monetized health impacts), and necessary application files (run control and code) are provided as part of this database to facilitate model comparison for current and future versions of these tools. For comparison, photochemical grid model annual average speciated PM_2.5_ output is provided for each emissions control scenario. Further, the input and output files for the Environmental Benefits and Mapping Community Edition (BenMAP-CE; https://www.epa.gov/benmap) system are also provided to allow for estimation of the monetized health impacts associated with each of these emission scenarios. Photochemical grid model output is from the Community Multiscale Air Quality (CMAQ) modeling system (https://www.epa.gov/cmaq) or the Comprehensive Air Quality Model with Extensions (CAMx; www.camx.com).

**Table 1:** Overview of the input and outputs for the reduced complexity models and photochemical models provided in this database.

Model	Emissions – Surface	Emissions – Elevated Point	Meteorology	Chemistry	Boundary Inflow	Air Quality Output
**CMAQ/CAMx**	Hourly year specific gridded 12 km	Hourly actual location and stack height	Hourly year specific gridded 12 km	Calculated during runtime (not input)	Hourly year specific gridded 12 km	Hourly gridded 12 km
**APEEP**	Annual county total	Annual county binned by stack release height	N/A	N/A	N/A	Annual county
**InMAP**	Annual year specific gridded 12 km	Annual actual location and stack height	Annual average year specific gridded 12 km	Annual average year specific gridded 12 km	N/A	Annual gridded 12 km
**EASIUR**	Annual year-specific gridded 36 km	Annual gridded 36 km binned by stack height	N/A	N/A	N/A	No air quality output

**Fig. 1:**
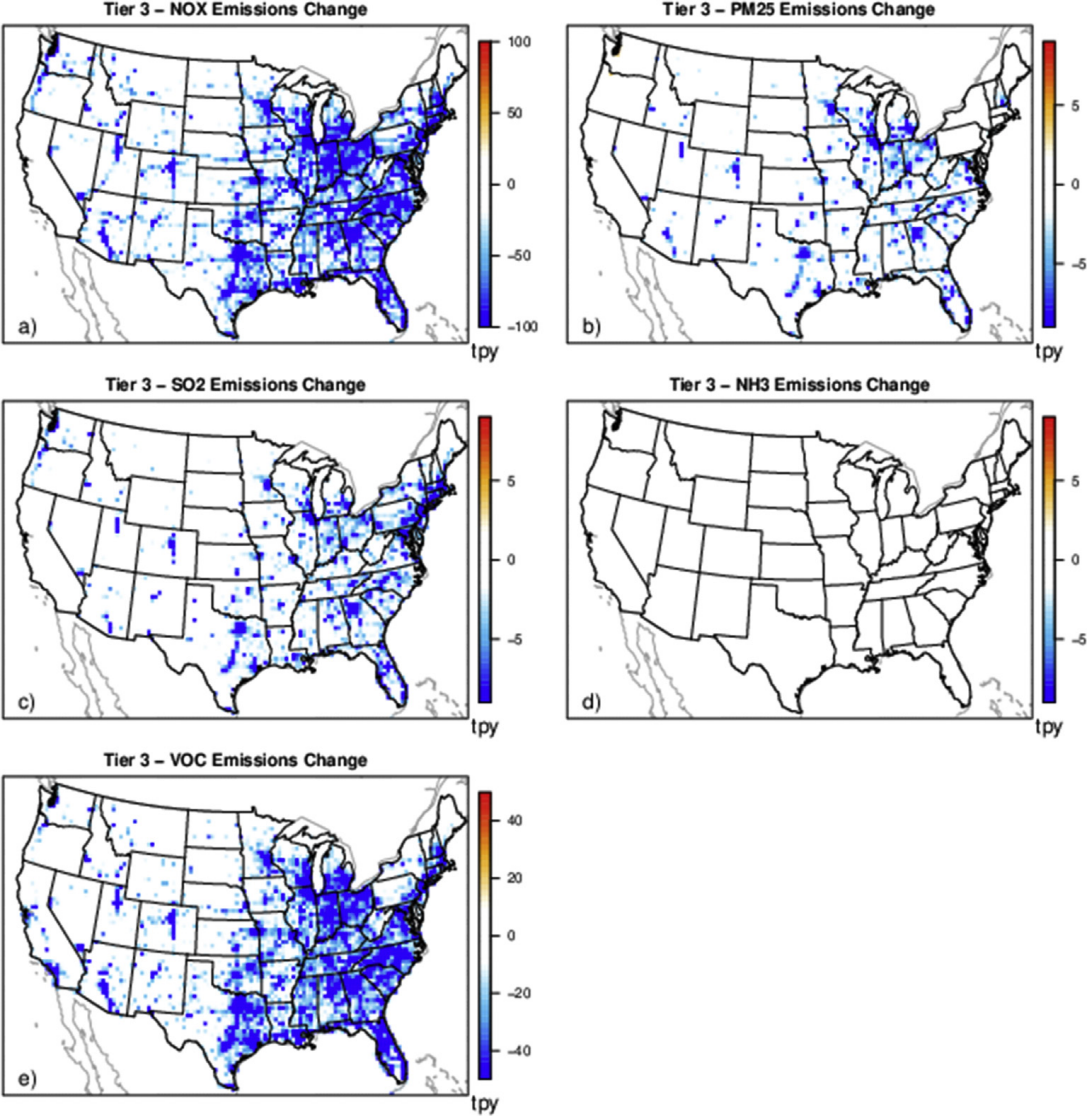
Change in annual emissions of a) NO_X_, b) primary PM_2.5_, c) SO_2_, d) NH_3_ and e) VOC for the Tier 3 scenario. Emissions have been gridded to 36 km sized cells. Cool colors show a decrease in emissions and warm colors represent an increase in emissions.

**Fig. 2:**
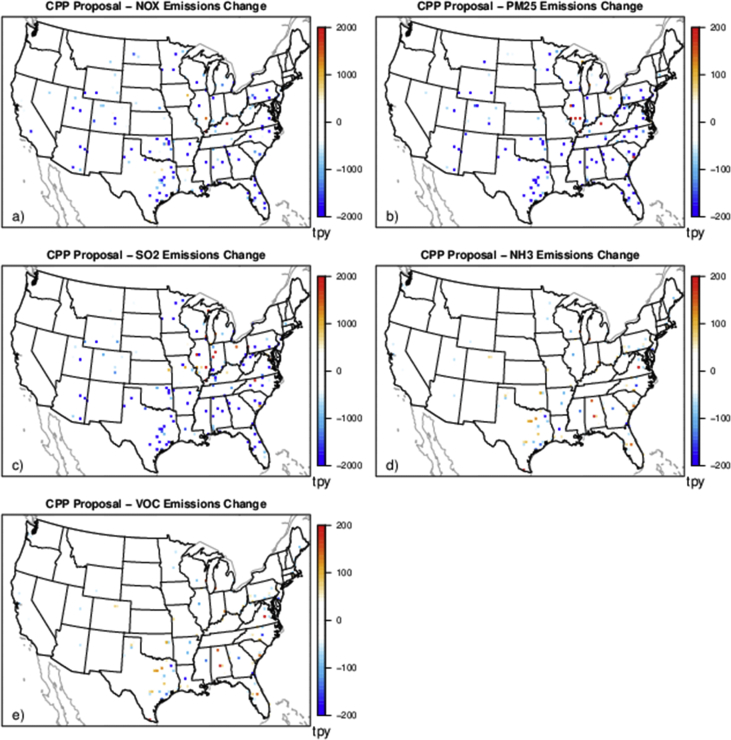
Change in annual emissions of a) NO_X_, b) primary PM_2.5_, c) SO_2_, d) NH_3_, and e) VOC for the Clean Power Plan proposal scenario. Emissions have been gridded to 36 km sized cells. Cool colors show a decrease in emissions and warm colors represent an increase in emissions.

**Fig. 3:**
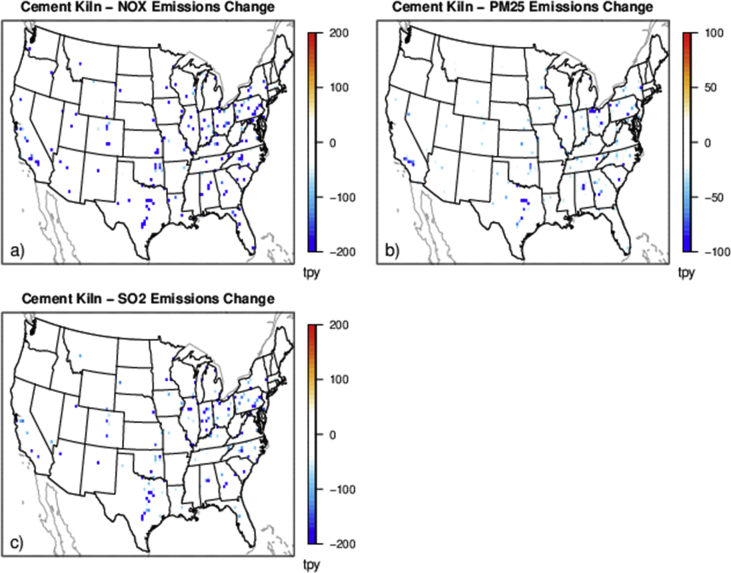
Change in annual emissions of a) NO_X_ top row, b) primary PM_2.5_, and c) SO_2_ for the hypothetical cement kiln emissions scenario. Cooler colors indicate a larger decrease in emissions.

**Fig. 4:**
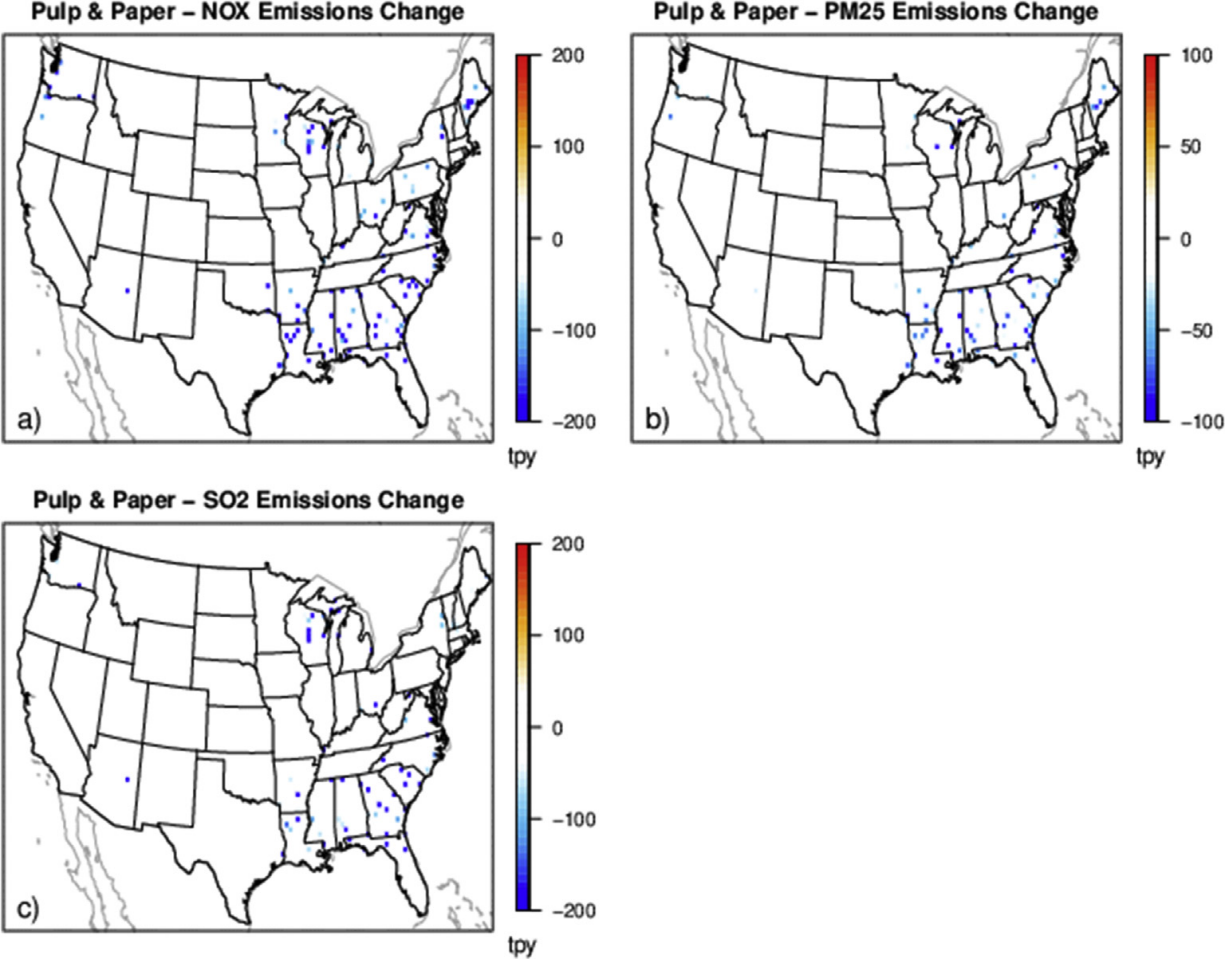
Change in annual emissions of a) NO_X_ top row, b) primary PM_2.5_, and c) SO_2_ for the hypothetical pulp and paper emissions scenario. Cooler colors indicate a larger decrease in emissions.

**Fig. 5:**
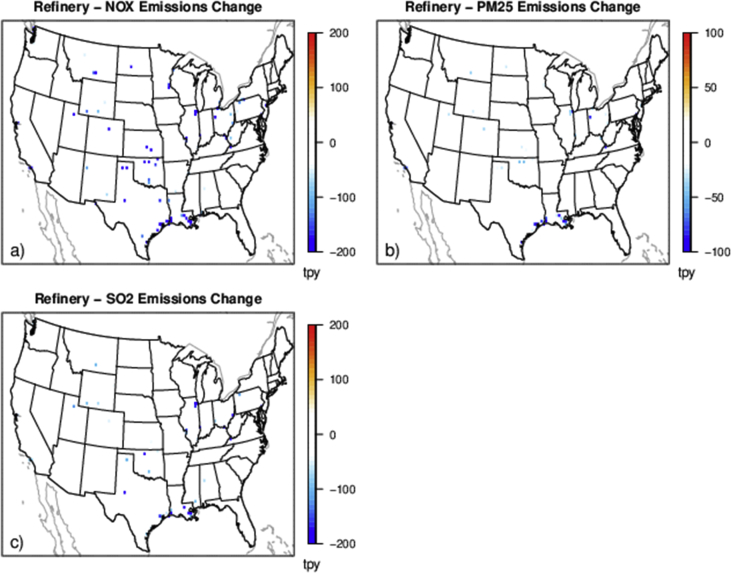
Change in annual emissions of a) NO_X_ top row, b) primary PM_2.5_, and c) SO_2_ for the hypothetical refinery emissions scenario. Cooler colors indicate a larger decrease in emissions.

The case studies simulate policies affecting emissions from various sources and sectors (e.g., power plants and onroad vehicles) and can provide a basis for evaluating the degree to which reduced complexity models represent air quality impacts and health outcomes resulting from emissions changes for a range of policy-relevant scenarios. There are five separate policy scenarios representing emissions reductions from various sources and sectors: electrical generating units (EGUs), onroad vehicles, cement kilns, refineries and pulp and paper facilities (Table 2). The national total changes in emissions for each of the case studies are provided in Table 2 and shown spatially for the onroad scenario in Fig. 1, EGU scenario in Fig. 2, and each of the industrial scenarios in Fig. 3, Fig. 4, Fig. 5. Table 3 provides a schematic showing how the model output species were mapped for comparability to make the comparison of total and chemically speciated components of particulate matter most consistent across modeling systems.

**Table 2:** Aggregated total annual emissions for 2007 and 2011 and annual emission reductions (tons) in directly emitted PM_2.5_ and PM_2.5_ precursors for each of the emission scenarios provided in this database.

Scenario	NOX	SO2	PM25	EC	NH3	VOC (anthropogenic)
**Reference 2007**	5,311,615	493,646	3,331,878	256,500	4,331,350	13,149,401
**Tier 3**	(348,467)	(13,132)	(8518)	(1332)	–	(181,840)
**Reference 2011**	9,540,403	2,871,999	4,668,823	373,798	4,416,704	15,132,910
**CPP proposal**	(424,237)	(426,529)	(63,192)	(2522)	(3306)	(10,094)
**Cement kiln**	(97,185)	(55,417)	(13,093)	(558)	–	–
**Pulp & paper**	(34,616)	(36,464)	(7197)	(278)	–	–
**Refinery**	(34,982)	(16,422)	(3932)	(424)	–	–

**Table 3:** Mapping model precursor emissions to model output and adjustments to modelled output for input to BenMAP. The empirical equation used to estimate particle bound water based on sulfate, nitrate, and ammonium concentrations is provided elsewhere [13].

Model	Emissions	Raw model output species	Input to BenMAP
CMAQ	SO_2_	ASO4I + ASO4J, ANH4I + ANH4J	(ANH4I + ANH4J + ASO4I + ASO4J) - (ANO3I + ANO3J × 0.29) + (PB_Water - (0.12 × ANO3I + ANO3J × 1.29))
CAMx	SO_2_	PSO4, PNH4	(PNH4 + PSO4) - (PNO3 * 0.29) + (PB_Water - (0.12 × PNO3 * 1.29))
InMAP	SO_2_	pSO4, pNH4	pSO4 * 1.37
APEEP	SO_2_	SO4 (assumed ammonium sulfate)	SO4 (ammonium sulfate)
CMAQ	NO_X_	ANO3I + ANO3J	(ANO3I + ANO3J) * 1.29 × 1.12
CAMx	NO_X_	PNO3	PNO3 * 1.29 × 1.12
InMAP	NO_X_	pNO3	pNO3 * 1.29
APEEP	NO_X_	NO3 (assumed ammonium nitrate)	NO3 (ammonium nitrate)
CMAQ	EC	AECI + AECJ	AECI + AECJ
CAMx	EC	PEC	PEC
InMAP	EC	PrimaryPM25 (only EC emissions)	PrimaryPM25
APEEP	EC	PM_25_Primary (only EC emissions)	PM_25_Primary

## 2 Experimental design, materials, and methods

### 2.1 Reduced complexity model application

For two of the reduced complexity tools (InMAP and EASIUR), run control files were constructed to clearly indicate what model options were selected so other users can reproduce the model predictions. InMAP and EASIUR have run control files that identify relevant input files (i.e., scenario-specific emissions) and the location and naming convention for scenario-specific output files. As distributed, APEEP does not include a run control file or standardized set of input or output files, only MATLAB files that users must modify to include emission scenario-specific information. A list of MATLAB subroutines, and the subroutines themselves, are provided so users understand the sequence of subroutine execution. Some of the APEEP code was modified to direct the modeling system to use particular emission input files with scenario-specific information and to automatically generate output files of the predicted air quality surface. The code also needed to be modified to reflect scenario-specific information (e.g., value of statistical life).

### 2.2 Emission scenarios

The 2014 Tier 3 Motor Vehicle Emission and Fuel Standards Final Rule (Tier 3) was selected as an onroad vehicles sector policy scenario [5]. The Tier 3 fuel and vehicle standards directly reduce emissions of NO_X_, volatile organic compounds (VOCs), PM_2.5_, and SO_2_. The emission inventories used include a 2030 future reference case (i.e. emissions representing a 2030 future year without any Tier 3 regulation) and a 2030 control case (i.e. emissions representing a 2030 future year with emissions expected under the Tier 3 regulation). The national total emissions reductions, between the 2030 reference (2030rg_ref_v5_07e) and control (2030rg_ctl_v5_07e) cases are provided in Table 2 and spatially in Fig. 1.

One control option from the 2015 Clean Power Plan (CPP) proposal [6] was selected (option 1S) for an EGU policy scenario. This CPP proposal was intended to implement greenhouse gas emission guidelines for existing fossil fuel fired EGUs with the goal of reducing carbon dioxide (CO_2_) emissions. Implementing the proposed CO_2_ emission guidelines was predicted to have ancillary emission reductions (i.e., co-benefits) of sulfur dioxide (SO_2_), nitrogen oxides (NO_X_), and directly emitted PM_2.5_, which would lead to lower ambient concentrations of PM_2.5_. The emission inventories include a 2025 future reference case (i.e. emissions representing a 2025 future year without any CPP regulation) and a 2025 control case (i.e. emissions representing a 2025 future year with emissions characteristic of the CPP option 1S scenario). The national total emissions changes between the projected future reference scenario (2025ef_v6_11g) and the future control scenario (2025ef_ghg-1S_v6_11g) are shown in Table 2 and spatially in Fig. 2.

Multiple industrial sector case studies were developed focused on sectors with unique geographic distributions of facilities: cement kilns, refineries, and pulp and paper facilities (Fig. 3, Fig. 4, Fig. 5). The Control Strategy Tool (CoST) program (https://www.epa.gov/economic-and-cost-analysis-air-pollution-regulations/cost-analysis-modelstools-air-pollution#control strategy tool) was applied to a 2025 future reference case for each sector and pollutant with a maximum emissions reduction algorithm to find the control technology option providing the maximum emissions reduction regardless of cost. The resulting relative change in emissions for each sector were aggregated nationally by the relevant North American Industry Classification System code.

For each of the hypothetical industrial sector policy scenarios, we applied a specific percentage of precursor emission reductions to all facilities in that sector for NO_X_, SO_2_ and PM_2.5_. The emissions inventories used the same 2025 future reference case as the CPP policy scenario (2025ef_v6_11g). For cement kilns, there was a respective 40%, 50% and 40% reduction of NO_X_, SO_2_ and primary PM_2.5_ applied to each source in the country. For refineries, there was a respective 40%, 15% and 15% reduction of NO_X_, SO_2_ and primary PM_2.5_ applied to each source. For pulp and paper facilities, there was a respective 20%, 35% and 25% reduction of NO_X_, SO_2_ and primary PM_2.5_ applied to each source. The industrial sectors national total emissions reductions are listed in Table 2.

### 2.3 Photochemical modeling benchmarks

CAMx version 6.10 was used to conduct the full-form air quality modeling for CPP proposal and the industrial sector policy scenarios and CMAQ version 4.7 was applied for the Tier 3 scenario. Both CMAQ and CAMx were applied with hourly emissions inputs for VOC, SO_X_, NO_X_, ammonia (NH_3_), and primary PM_2.5_. Both CMAQ and CAMx were applied with gridded low-level emissions, location-specific elevated point emissions sources, and gridded meteorology input files. Emissions and meteorological inputs to CMAQ are based on netCDF file format and CAMx are a structured binary format. Hourly 2007 meteorological inputs are provided for CMAQ for the Tier 3 related emissions and hourly 2011 meteorological inputs are provided for CAMx for CPP proposal and the industrial sector scenarios.

### 2.4 Emissions input files

The user-specified emissions input required for running InMAP is a shapefile or set of shapefiles containing annual total emissions of VOCs, SO_2_, NO_X_, VOC, NH_3_, and primary PM_2.5_ (not chemically speciated). Photochemical model emissions inputs files were converted to shapefile format for use in InMAP. Shapefiles were created for gridded annual total non-point emissions and separate shapefiles with location specific annual point source emissions. All emissions are in tons per year. Separate shapefiles of gridded 2D emissions were created that include 1) anthropogenic emissions and 2) biogenic emissions. The biogenic emissions included both biogenic and wildland fire. A third shapefile contained all anthropogenic point sources with location and stack release information.

APEEP (version 2 and 3) uses annual county total emissions as input data. EPA calculated county level annual total emissions of NH_3_, NO_X_, SO_2_, primarily emitted PM_2.5_ (not chemically speciated), VOC (not chemically speciated) from anthropogenic sources, and VOC (not chemically speciated) from biogenic sources for each scenario. Emissions files are provided as text-format comma delimited files with emission rates for each U.S. county included in the APEEP source-receptor matrix. No emissions were included from Canada, Mexico, or offshore locations, as the APEEP model does not have relevant source-receptor relationships. All emissions were in units of tons per year. Separate files were generated for each policy scenario including emissions by varying release height: 1) “ground” level (all non-point) emissions, 2) “low” level, or point sources with effective stack height less than 200 m, 3) “medium” level, or point sources with effective stack height between 200 m and 500 m, 4) “tall” level, or point sources with effective stack height greater than 500 m, and 5) “new tall”, or point sources with effective stack height greater than 500 m that were not part of the original source-receptor matrix and added later. Emissions for “new tall” point sources were included as multiple sources per county that added up to the county total. Not all U.S. counties are represented in APEEP's “tall” stack source-receptor matrix. In these situations, “tall” stack emissions were put into the “medium” stack emissions source-receptor matrix so they would be represented in the model simulation.

EASIUR emissions input files were generated for each of the scenarios matching the EASIUR 36 km grid cell resolution domain covering the contiguous U.S. and then converted to ascii text format (comma delimited files). Each file contains gridded annual emissions of NH_3_, NO_X_, SO_2_, and primarily emitted PM_2.5_ (not chemically speciated). All grid cells that are part of the EASIUR 36 km domain were included in each file. Where a grid cell did not contain emissions, a 0 value was assigned to each species so that each grid cell has a record. Separate files for each scenario are provided by varying emission release height: 1) gridded (all non-point) emissions, 2) point sources with actual stack height less than 150 m, 3) point sources with actual stack height between 150 and 300 m, and 4) point sources with actual stack height greater than 300 m.

### 2.5 Meteorological and chemical input files

InMAP requires a single netCDF format input file containing 3D annual average meteorology, air quality, and deposition information. This input file includes spatially explicit annual averages of wind vectors, eddy diffusivity and convective transport coefficients (annual average coefficients calculated using temporally explicit wind speed, temperature, pressure, friction velocity, boundary layer height, and heat flux information), dry and wet deposition rates of various pollutants (annual average rates calculated using temporally explicit wind speed, land cover, stability, and precipitation information), gas/particle phase partitioning for pollutants, and parameters relevant to the calculation of emissions plume rise (annual averages of scalar windspeed; temperature; and two parameters related to atmospheric stability).

InMAP is distributed with a netCDF input file for optional use that has chemical and meteorological parameters based on values derived from a simulation using the WRF-Chem Eulerian model [7] applied with emissions from the 2005 National Emissions Inventory (NEI [8]). Alternatively, users can use annual meteorological and photochemical model simulations to develop their own annual average meteorology/chemistry/deposition input file. The input filed provided in this dataset was generated using output from 2007 WRF and CMAQ simulations for the onroad mobile emissions scenario and from 2011 WRF and CAMx simulations for the EGU and industrial sector emissions scenarios using the conversion utility distributed with InMAP (https://godoc.org/github.com/spatialmodel/inmap/inmaputil#ConfigData.Preproc). The 2007 WRF/CMAQ and 2011 WRF/CAMx outputs were obtained from previously available model simulations that are described in Refs. [5,9] respectively. The conversion utility was updated to work with WRF and CMAQ/CAMx since the distributed version only had compatibility with WRF-Chem and GEOS-CHEM output.

APEEP, EASIUR, and SA-BPT do not accept user-supplied meteorological input files although the formulation for these models was developed using meteorological parameters. EASIUR and SA-BPT were both parameterized based on model simulations that used 2005 meteorology [3,4]. APEEP contains source-receptor matrices for the formation and transport of particulate matter to produce annual means which was generated by the Gaussian model using climatological meteorology [2,10].

### 2.6 Air quality model output

Annual average PM_2.5_ surfaces output for each of the emissions scenarios by each of the reduced complexity and photochemical grid models are provided to allow for inter-comparison and benchmarking to ensure model inputs were correctly applied. Files are available for each modeling system and each emission scenario. The APEEP model directly outputs county level total PM_2.5_. For InMAP, the 12 km gridded total PM_2.5_ was estimated by summing PM_2.5_ chemical components: nitrate, sulfate, ammonium, primary PM_2.5_, and secondary organic aerosol. Full-scale hourly photochemical model PM_2.5_ chemical component output was aggregated to annual average. Each reduced complexity model predicts annual average PM_2.5_ so no temporal aggregation was necessary. InMAP output are provided as shapefiles, APEEP as comma-delimited text files, and the annual aggregated photochemical model output as netCDF based files.

### 2.7 BenMAP-CE

Model predicted annual PM_2.5_ was converted for input to BenMAP-CE [11]. BenMAP input files were generated for each emissions scenario and model. Following the approach typically used in past benefits assessments, photochemical model estimated annual PM_2.5_ was adjusted with ambient speciated PM_2.5_ measurements from routine surface monitor networks using a statistical technique part of EPA's Software for Model Attainment Test-Community Edition to minimize areas of extreme over or under prediction tendency [12,13]. Table 3 shows how precursor emissions relate to raw model output species and adjustments made to those species for input to BenMAP to estimate monetized health benefits associated with specific precursors.

InMAP and photochemical model output were converted to the comma delimited format required for input to BenMAP with annual PM_2.5_ gridded to match the 12 km sized grid cell model domain. The APEEP inputs to BenMAP are county specific rather than gridded. BenMAP input and output files for each of the modeling systems part of this analysis and each of the emissions scenarios are provided as part of this database. All BenMAP outputs are text files with county specific information.

### 2.8 Estimating monetized health benefits for SA-BPT and EASIUR

The BenMAP tool estimates monetized health damages associated with PM_2.5_ changes in each county. EASIUR and SA-BPT provide this information as a look-up table rather than by producing intermediate air quality estimates. SA-BPT estimates nationally aggregated monetized health benefits by multiplying a change in PM_2.5_ precursor emissions by pre-computed marginal benefits estimated for specific sectors. Relevant SA-BPT sectors used here include onroad mobile, EGUs, cement kilns, refineries, and pulp and paper facilities [4]. The sector specific benefit-per-ton values used for this assessment were adjusted to reflect a value of statistical life (VSL) projected to 2015. Nationally aggregated emissions changes by precursor are provided for use with the SA-BPT in Table 2.

A program was developed to match the EASIUR grid-cell, stack height (surface or elevated release), and precursor emissions specific monetized health damage estimates with scenario-specific emissions information. The program adjusted the aggregated monetized health damages estimated by EASIUR to reflect a 2015 VSL.
